# Vessel Density and Vessel Tortuosity Quantitative Analysis of Arteritic and Non-arteritic Anterior Ischemic Optic Neuropathies: An Optical Coherence Tomography Angiography Study

**DOI:** 10.3390/jcm9041094

**Published:** 2020-04-12

**Authors:** Luisa Pierro, Alessandro Arrigo, Emanuela Aragona, Michele Cavalleri, Francesco Bandello

**Affiliations:** Department of Ophthalmology, Scientific Institute San Raffaele, Vita-Salute University, 20132 Milan, Italy; pierro.luisa@hsr.it (L.P.); aragona.emanuela@hsr.it (E.A.); cavalleri.michele@hsr.it (M.C.); bandello.francesco@hsr.it (F.B.)

**Keywords:** arteritic anterior ischemic optic neuropathy, Non-arteritic anterior ischemic optic neuropathy, optical coherence tomography (OCT), quantitative optical coherence tomography angiography (OCTA), vessel density, vessel tortuosity

## Abstract

The aim of this study was to perform quantitative optical coherence tomography angiography (OCTA) assessment of arteritic and non-arteritic anterior ischemic optic neuropathies (AION; NAION). The study was designed as an observational, cross-sectional case series. All patients underwent complete ophthalmologic evaluation including LogMAR best-corrected visual acuity (BCVA), structural optical coherence tomography (OCT) and OCTA images, and dye-based angiography. Retinal nerve fiber layer (RNFL) thickness was obtained from structural OCT, and vessel density (VD) and vessel tortuosity (VT) were measured for each optic nerve head vascular plexus. After selecting the quadrants showing visual field defects, measured by Humphrey 30.2 perimetry (Zeiss Meditec, Dublin, CA, USA), we assessed the correlation between the localization of visual field defects and the quadrants showing impairments of RNFL, VD, and VT. Thirty naïve AION patients (15 arteritic AION (AAION) and 15 non-arteritic AION (NAION)) were included. LogMAR BCVA was 0.6 ± 0.2 for AAION and 0.3 ± 0.3 for NAION (*p* < 0.01). AAION and NAION eyes showed significant differences in terms of visual field involvement as well as VD and VT values, with remarkably worse alterations affecting AAION eyes. VD values perfectly matched with the quadrants showing RNFL and visual field defects. On the contrary, VT resulted remarkably decreased in all the quadrants, with even worse values in the quadrants showing RNFL and visual field alterations. The present study showed that AAION eyes are more injured than NAION ones. VD represents a good parameter for the detection of the main site on vascular impairment. Remarkably, VT resulted in a more sensitive parameter for the quantitative detection of blood flow impairment in AION disease.

## 1. Introduction

Anterior ischemic optic neuropathies (AION) represents a set of diseases affecting the anterior portion of the optic nerve, distinguished as arteritic (AAION) and non-arteritic (NAION) [1]. NAION is the most common disorder affecting the optic nerve, except for glaucoma. The pathogenesis is not fully understood, although previous evidence suggests a role of a transitory optic nerve hypoperfusion, leading to the onset of the ischemia [1]. On the other hand, AAION represents an optic nerve disease strongly associated with giant-cell arteritis (GCA), thus having the vasculitis as the key factor of its pathogenesis [1]. Moving beyond the starting factor leading to the onset of the disease, both NAION and AAION are characterized by the concomitant presence of optic nerve ischemia and inflammation, both contributing to the onset and progression of typical symptoms, such as visual acuity reduction and visual field defects [2]. The diagnostic workup of AION benefits from the combined use of fluorescein angiography (FA) and non-invasive multimodal imaging [2]. In this context, structural optical coherence tomography (OCT) and OCT angiography (OCTA) provided very detailed information regarding the structural and vascular impairment of the optic nerve [3,4]. Swept source OCT technology was a major advance in terms of imaging techniques; indeed, the fast acquisition speed (up to 100,000 A-scans per second) and the high-resolution (up to 8 µm optical function) markedly improved the retinal morphological details achievable in vivo in the human retina [5,6]. In particular, the vessel density decrement of the optic nerve head vascular plexa is evident at the level of the deep capillary plexus and the radial peripapillary capillaries have been reported. It is worth noting that the adoption of OCTA technology allows one to study microstructural features of the retinal vascular network without the adoption of dye, and with enhanced resolution of smaller retinal vessels.

The main aim of the present study is to investigate in deep the quantitative OCTA features of AAION and NAION to assess possible differences in terms of vascular impairment.

## 2. Materials and Methods

The study was set as observational and cross-sectional. Both patients affected by NAION and AAION were recruited from our center (IRCCS San Raffaele Hospital Milan, Italy). Inclusion criterion was the confirmed diagnosis of the first acute episode of NAION or AAION. In particular, the diagnosis of AAION was definitively confirmed by temporal artery biopsy, revealing GCA. NAION diagnosis was confirmed with the following criteria: sudden and painless vision loss, optic disc edema with or without superficial hemorrhages at the optic disc border and adjacent retina on fundus examination, and visual field defects suggestive of NAION. Exclusion criteria were: history of glaucoma or retinal diseases, any other optic neuropathy, previous episode of NAION or AAION, ophthalmic surgery in the last six months, or any other ocular or systemic disorder potentially affecting the results of the study. A control group made up of healthy age- and sex-matched subjects was included as well. NAION or AAION diagnosis was confirmed by means of FA examination (Spectralis HRA2, Heidelberg Engineering, Heidelberg, Germany). The entire study was approved by the ethical committee of IRCCS San Raffaele Hospital Milan, and it was conducted in accordance with Helsinki declaration. All subjects/patients gave their informed consent for inclusion before they participated in the study.

All the patients underwent complete ophthalmologic examination including best corrected visual acuity (BCVA), slit-lamp evaluation of anterior and posterior segments, and tonometry.

Structural OCT and OCTA (Swept Source OCT DRI Topcon Triton; Topcon Corporation, Tokyo, Japan) images were acquired. From structural OCT, we extracted the quantitative measure of retinal nerve fiber layer (RNFL) thickness. OCTA images included 3 × 3 mm and 4.5 × 4.5 mm high resolution scans, centered on the optic nerve head (wavelength 1050 nm; 100,000 A-Scans per second; in-depth resolution digital 2.6 µm and optical function 8 µm). We considered only high-quality images, assessed by Topcon image quality index (≥70) [7]. The automatic segmentation of capillary plexa (radial peripapillary capillaries (RPC), superficial (SCP), deep (DCP) and choriocapillaris (CC)) was obtained from OCTA acquisitions. Each reconstruction was carefully inspected by an expert ophthalmologist (L.P.) and manually corrected if necessary. All reconstructions were exported in the .tiff format and loaded in ImageJ software (https://imagej.net/Welcome). In-house scripts were built in order to calculate vessel density (VD) and vessel tortuosity (VT) parameters. VD was calculated after image binarization was obtained by applying a mean threshold and then by calculating the percentages of white pixels compared to black ones. VT was calculated after applying the “skeletonize” function to all reconstructions, enabling each vessel to be considered as a line. Strahler analysis was applied in order to identify each root better and to reduce possible bias (https://imagej.net/Strahler_Analysis#Root_Detection) [8]. Then, we calculated the Euclidean distance on each line corresponding to vessels in order to measure the kind of path followed by each vessel: either a line or a curve. We performed a sub-analysis in order to assess the relationship between imaging findings and visual field defects. We separately considered the quadrants showing significant thinning of RNFL, as well as significant changes of VD and VT parameters, in this case only considering RPC and SCP, from the quadrants showing unremarkable changes, with respect to controls. Then, after selecting the quadrants showing visual field defects, measured by Humphrey 30.2 perimetry (Zeiss Meditec, Dublin, CA, USA), we assessed the correlation between the localization of visual field defects and the quadrants showing impairments of RNFL, VD, and VT (Tau–Kendall correlation test). The statistical analysis of the quantitative parameters considered, as compared with control values, was performed using the one-way Analysis of variance (ANOVA) test with Bonferroni correction for multiple comparisons (IBM SPSS Statistics V.20.0, International Business Machine Corporation, Armonk, NY, USA). The statistical significance was set at *p* < 0.05.

## 3. Results

In the present study, 15 eyes of 15 AAION patients (eight males; mean age 48.5 ± 10.2 years), 15 eyes of 15 NAION patients (nine males; mean age 46.9 ± 12.5 years) and 15 healthy controls (eight males; mean age 47.5 ± 8.3 years) were included into the analysis. Both AAION and NAION contralateral and apparently normal eyes were included and separately considered. Ten additional AAION eyes were excluded (five for bilateral involvement, five for very poor-quality images) as well as five additional NAION eyes (three for uncontrolled arterial hypertension, two for very poor-quality images).

The mean LogMAR BCVA was 0.6 ± 0.2 for AAION and 0.3 ± 0.3 for NAION (*p* < 0.01). Both contralateral eyes and healthy controls showed LogMAR BCVA of 0.0 ± 0.0. LogMAR BCVA, which was significantly worse in affected eyes than in contralateral and control eyes (*p* < 0.01).

Both AAION and NAION eyes ophthalmoscopically showed an optic nerve head edema, which was more prominent in AAION than NAION eyes. Contralateral eyes showed unremarkable changes. RNFL was significantly reduced both in AAION (74 ± 15 µm) and NAION eyes (85 ± 16 µm) compared to controls (115 ± 10 µm; *p* < 0.01).

**Figure 1:**
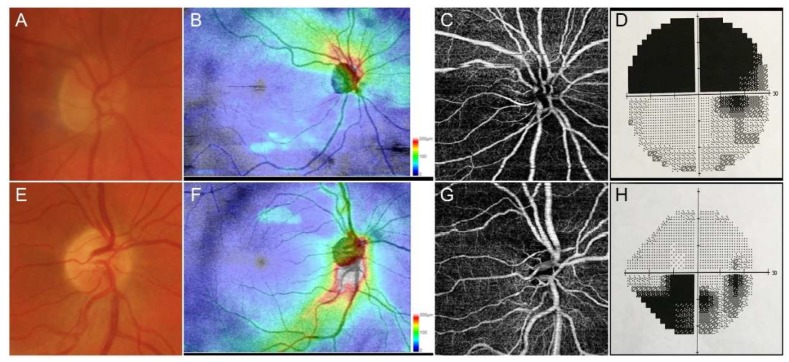
Representative cases of anterior ischemic optic neuropathies. Anterior ischemic optic neuropathies (AAION) case showed a marked involvement of optic nerve head (**A**), with a strong damage of retinal nerve fiber layer (RNFL) (**B**), together with sectorial optical coherence tomography angiography (OCTA) perfusion impairment (**C**) and superior visual field defects (**D**). Non-anterior ischemic optic neuropathies (NAION) cases showed a less extensive optic nerve head (**E**) and RNFL (**F**) involvement, with a well-detected OCTA perfusion damage (**G**) and circumscribed visual field defect (**H**).

OCTA quantitative analysis revealed significant differences between patients and controls (Table 1). Representative cases of AAION and NAION are shown in Figure 1. In particular, AAION and NAION eyes showed significant differences in terms of VD values for RPC and SCP (*p* < 0.01), whereas DCP and CC was unremarkable (*p* > 0.05). On the contrary, VT values were significantly lower in AAION than NAION for SCP, DCP, and RPC (*p* < 0.01). VD and VT values were significantly lower both in AAION and NAION eyes compared to contralateral and control eyes (*p* < 0.01). Furthermore, no significant changes were detected when comparing contralateral eyes comparing with controls.

**Figure 2:**
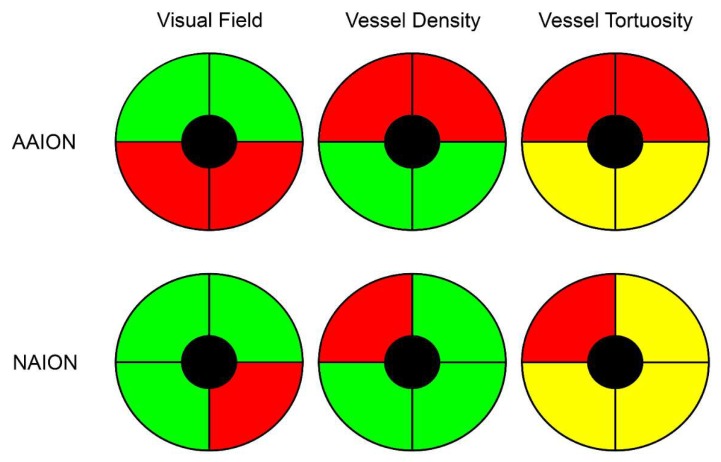
Schematic representation of vessel density and vessel tortuosity defects in anterior ischemic optic neuropathies. Although visual field defects were more pronounced in AAION than NAION, both diseases showed a perfectly matched vessel density reduction (red quadrants), with unremarkable changes in the other sectors (green quadrants), compared to healthy controls. On the other hand, vessel tortuosity registered the worst values in the affected quadrants (red quadrants), but statistically significant lower values were also found in the unaffected quadrants (yellow quadrants), with respect to healthy controls.

**Figure 3:**
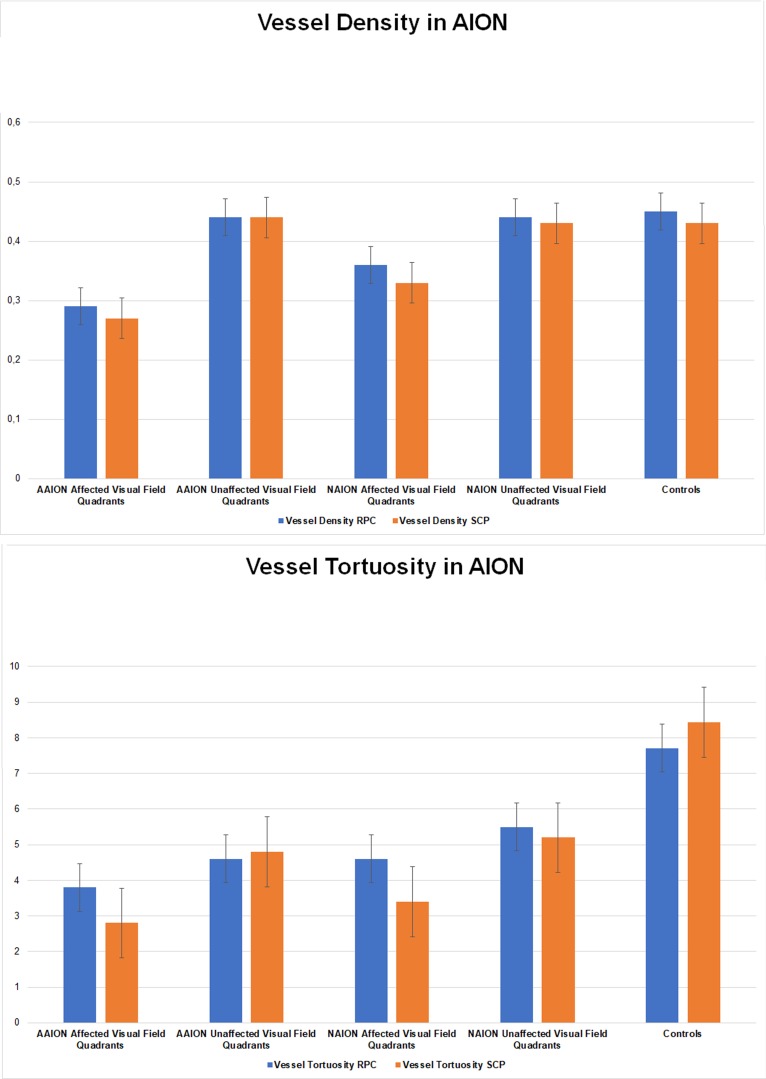
Bar graphs of vessel density and vessel tortuosity values in affected and unaffected quadrants of AAION and NAION patients. (**A**). Vessel Density in AION, (**B**). Vessel Tortuosity in AION.

The correlation analysis showed very good matching between quadrants affected by RNFL and VD decrease and visual field defects (Tau–Kendall coefficient 0.89 and 0.85 respectively; *p* < 0.01), whereas no correlation was found between VT and the remaining parameters (RNFL, visual field defects, VD quadrants; *p* > 0.05). A schematic representation of the detected phenomenon is shown in Figure 2. In particular, the sub-analysis showed unremarkable changes of RNFL and VD values in the unaffected quadrants, whereas VT was reduced in all optic nerve head quadrants; it is worth noting the statistical significant difference between the VT values of the quadrants showing unremarkable visual field defect and the VT values of the controls (*p* < 0.01). The resumed values are reported in Table 2 and graphically shown in Figure 3.

## 4. Discussion

AION represents irreversible causes of optic nerve damage. Whereas clinical presentation is often worse than the final outcome due to the resolution of the acute optic nerve edema, AION leads to permanent visual field defects [1]. The recently introduced non-invasive OCTA technique provides useful information regarding the vascular impairment occurring in AION. In particular, remarkable perfusion reductions have been described; these appear to be often localized and well-matching with the visual field defects [7,8,9,10]. This is somehow expected, as the co-existing presence of optic disc edema and hypoperfusion leads to RNFL damage and then visual field alterations. When looking at the literature, most of the OCTA studies have focused on NAION than AAION [9,10,11]. Furthermore, to the best of our knowledge, the entity of perfusion defects occurring in AAION and NAION has not been studied in depth to highlight possible differences between these two conditions.

In the present study, we analyzed eyes with new diagnosis of AAION or NAION and we included also the contralateral unaffected eye, in order to analyze eventual possible differences with respect to healthy controls. We confirmed the statistically significant vascular impairment occurring in AAION and NAION eyes. It is worth noting that AAION eyes showed significantly worse optic nerve hypoperfusion than NAION eyes, which may be explained by the higher amount of optic disc swelling characterizing AAION [1]. Contralateral eyes of both categories of patients showed unremarkable vascular changes compared to healthy controls. As previously described, OCTA findings show very good matching with RNFL and visual field damages, confirmed by correlation analyses. In the present study, we reported a different behavior between vessel density and vessel tortuosity parameters. In particular, vessel density showed significant reductions only in quadrants corresponding with visual field defects, whereas vessel tortuosity impairment was statistically significant both in quadrants corresponding to visual field defects and in quadrants without remarkable perimetric alterations. The amount of vessel tortuosity decrease was statistically worse in the affected quadrants than in the unaffected ones. This phenomenon was not observed for vessel density and may explain the lack of vessel tortuosity correlations with RNFL and visual field impairments. Taking into consideration the overall distribution of optic disc swelling in all the quadrants, based on our data and on previous findings [12,13], vessel tortuosity might be considered a more sensitive parameter for the quantification of blood flow impairment occurring in AAION and NAION. Also, in the sub-analysis, AAION showed worse vessel density and vessel tortuosity values than NAION. Based on our findings, we may advance the hypothesis that the combined influence of optic disc edema and ocular blood flow reduction characterizing both AAION and NAION, might determine an overall perfusion reduction of the optic nerve head, which can be well-detected by VT in all the optic nerve quadrants. The strongest perfusion reduction might be responsible also of an irreversible vascular damage; in this case not affecting the overall optic nerve head, but just selective quadrants. This irreversible alteration might be the responsible for VD reduction in the early phase, then RNFL and visual field alterations in later stages of both diseases. From this point of view, the combined use of VT and VD might cover an important role in terms of the cross-sectional evaluation of optic nerve damage, performed already during the first visit. Moreover, these two parameters, with particular regards to VT, might also be important in terms of the prognostic evaluations of AION patients. For this reason, further studies including both AAION and NAION patients are warranted to confirm our hypotheses.

Our study had some limitations. The first was related to the possible artifacts affecting OCTA images [14]. With respect to VT parameter, this represents a novel quantitative parameter which may be interpreted as an indirect measure of the amount of perfusion [13,15]. Although it is based on the proper reconstruction of the vascular network, it is potentially artifact prone. On the other hand, since we used only high quality images and the calculation of VT strictly depends on the geometrical properties of continuous linear signal retinal capillaries [13,15] with poor or null influence of spurious artifact signal, it may be considered a reliable quantitative measure of the perfusion features of the retinal vascular network. Moreover, although we selected only high-quality images, we cannot exclude the possible influence of optic disc swelling on the detection of blood flow signal. Furthermore, the limited number of cases does not allow us to reach definite conclusions, and further longitudinal studies are needed to assess the differences regarding vascular impairment in AAION and NAION.

In conclusion, our study introduced a new parameter, namely vessel tortuosity, which was more sensitive to blood flow perfusion defects occurring at early stages of AAION and NAION. OCTA quantitative analysis showed more vascular impairment in AAION than NAION, and a very strong correlation with RNFL and visual field damage. Further larger studies are needed to establish possible cutoff quantitative values which might have a role for the differential diagnosis between AAION from NAION, as well as for the better assessment of vascular and structural impairment of the optic nerve head.

## Figures and Tables

**Table 1:** Quantitative optical coherence tomography angiography (OCTA) analysis in anterior ischemic optic neuropathies.

**Quantitative Analysis**														
**Group**	**VD RPC**		**VD SCP**		**VD DCP**		**VD CC**		**VT RPC**		**VT SCP**		**VT DCP**	
	**Mean**	**STD**	**Mean**	**STD**	**Mean**	**STD**	**Mean**	**STD**	**Mean**	**STD**	**Mean**	**STD**	**Mean**	**STD**
AION	0.37	0.01	0.36	0.02	0.28	0.01	0.47	0.03	4.56	0.38	3.81	0.38	4.43	0.30
NAION	0.40	0.01	0.39	0.02	0.28	0.01	0.48	0.03	5.11	0.42	4.27	0.42	4.97	0.34
Contralat. AION	0.44	0.01	0.42	0.01	0.39	0.01	0.52	0.03	7.77	0.23	8.36	0.30	6.94	0.24
Contralat. NAION	0.44	0.00	0.42	0.01	0.39	0.02	0.53	0.02	7.61	0.20	8.37	0.42	7.08	0.25
Controls	0.45	0.01	0.43	0.01	0.40	0.02	0.55	0.03	7.71	0.39	8.43	0.26	7.06	0.23
***p*-values**	**VD RPC**		**VD SCP**		**VD DCP**		**VD CC**		**VT RPC**		**VTSCP**		**VT DCP**	
AION vs. NAION	<0.01		<0.01		>0.05		>0.05		<0.01		<0.01		<0.01	
AION vs. Controls	<0.01		<0.01		<0.01		<0.01		<0.01		<0.01		<0.01	
NAION vs. Controls	<0.01		<0.01		<0.01		<0.01		<0.01		<0.01		<0.01	
AION vs. Contralat. AION	<0.01		<0.01		<0.01		<0.01		<0.01		<0.01		<0.01	
NAION vs. Contralat. NAION	<0.01		<0.01		<0.01		<0.01		<0.01		<0.01		<0.01	
Contralat. AION vs. Controls	>0.05		>0.05		>0.05		>0.05		>0.05		>0.05		>0.05	
Contralat. NAION vs. Controls	>0.05		>0.05		>0.05		>0.05		>0.05		>0.05		>0.05	

**Table 2:** Anterior ischemic optic neuropathies (AAION) vs. non-anterior ischemic optic neuropathies NAION OCTA quantitative sub-analysis.

**AAION vs. NAION Sub-Analysis**						
**Parameter**	**AAION**		**NAION**		**Controls**	
	**Affected Visual Field Quadrants**	Unaffected Visual Field Quadrants	Affected Visual Field Quadrants	Unaffected Visual Field Quadrants		
**Vessel Density RPC**	0.29 ± 0.04	0.44 ± 0.01	0.36 ± 0.01	0.44 ± 0.01	0.45 ± 0.01	
**Vessel Density SCP**	0.27 ± 0.04	0.44 ± 0.02	0.33 ± 0.04	0.43 ± 0.01	0.43 ± 0.01	
**Vessel Tortuosity RPC**	3.8 ± 0.4	4.6 ± 0.8	4.6 ± 0.7	5.5 ± 0.7	7.71 ± 0.39	
**Vessel Tortuosity SCP**	2.8 ± 0.8	4.8 ± 0.8	3.4 ± 0.5	5.2 ± 0.8	8.43 ± 0.26	
***p*-values**			**Vessel Density RPC**	**Vessel Density SCP**	**Vessel Tortuosity RPC**	**Vessel Tortuosity SCP**
**AAION**	Affected vs. Unaffected Quadrants		*p* < 0.01	*p* < 0.01	*p* < 0.01	*p* < 0.01
	Affected Quadrants vs. Controls		*p* < 0.01	*p* < 0.01	*p* < 0.01	*p* < 0.01
	Unaffected Quadrants vs. Controls		*p* > 0.05	*p* > 0.05	*p* < 0.01	*p* < 0.01
**NAION**	Affected vs. Unaffected Quadrants		*p* < 0.01	*p* < 0.01	*p* < 0.01	*p* < 0.01
	Affected Quadrants vs. Controls		*p* < 0.01	*p* < 0.01	*p* < 0.01	*p* < 0.01
	Unaffected Quadrants vs. Controls		*p* > 0.05	*p* > 0.05	*p* < 0.01	*p* < 0.01
**AAION vs. NAION**	Affected vs. Affected Quadrants		*p* < 0.01	*p* < 0.01	*p* < 0.01	*p* < 0.01
	Unaffected vs. Unaffected Quadrants		*p* > 0.05	*p* > 0.05	*p* < 0.01	*p* < 0.01
